# Natural progression of meningeal lymphatic dysfunction in APP/PS1 mice creates a critical window for Alzheimer’s disease intervention

**DOI:** 10.55730/1300-0144.6116

**Published:** 2025-10-26

**Authors:** Zilong SHEN, Xibin ZHOU, Lin HE, Mingjie WU, Chunxiang ZHOU

**Affiliations:** Division of Shanghan Lun, Department of Classical Chinese Medicine, School of Chinese Medicine, Nanjing University of Chinese Medicine, Nanjing, China

**Keywords:** Meningeal lymphatic vessels, deep cervical lymph nodes, APP/PS1 mice, Alzheimer’s disease, Aβ

## Abstract

**Background/aim:**

Meningeal lymphatic vessels (mLVs) facilitate the clearance of toxic metabolites like amyloid-beta (Aβ) from the central nervous system. Dysfunction in MLVs is implicated in Alzheimer’s disease (AD). However, current knowledge relies on exogenous intervention models that fail to capture spontaneous mLV decline during AD progression. In this study, we investigated the age-dependent correlation between mLV/deep cervical lymph node (dCLN) dysfunction and Aβ pathology in APP/PS1 mice under noninterventional conditions.

**Materials and methods:**

APP/PS1 and wild-type (WT) mice at 3, 6, and 9 months of age were evaluated. Cognitive function was tested using the Morris water maze. mLV/dCLN drainage was assessed by intracisternal Texas Red dextran 3 injection. Lymphatic structure/function and Aβ pathology were analyzed via immunohistochemistry, immunofluorescence, and tracer penetration.

**Results:**

APP/PS1 mice developed significant cognitive deficits at 6 and 9 months. Aβ plaques emerged at 6 months and progressed by 9 months in APP/PS1 mice, but were absent in controls. At 6 months, APP/PS1 mice had reduced tracer drainage in mLVs/dCLNs, decreased LYVE-1 expression, and impaired tracer penetration in the hippocampus/cortex compared to WT mice.

**Conclusion:**

Lymphatic functional decline starts at 6-months old, providing a critical timeframe for early AD intervention. Our findings underscore the value of the APP/PS1 model for studying lymphatic clearance mechanisms in AD.

## 1. Introduction

In 2015, Louveau et al. [1] made a breakthrough in confirming the key role of human meningeal lymphatic vessels (mLVs) in the drainage of the central nervous system. This discovery challenged traditional understandings and showed that the glymphatic system and mLVs in the brain are the primary pathway for clearing toxic metabolites such as amyloid-beta (Aβ) [2]. mLV dysfunction leads to abnormal Aβ deposition and is closely related to the neurodegenerative pathology of Alzheimer’s disease (AD) [3]. This means that mLVs may be a new target for AD treatment.

Extracellular amyloid plaques, a typical pathological hallmark of AD, are mainly composed of aggregated Aβ peptides [4]. Currently, most studies use transgenic mice carrying *APP*/*PSEN1* mutant genes (such as the APP/PS1 model) to simulate the AD process [5, 6]. This model shows that plaques grow rapidly at 6 months of age [7], accompanied by synaptic transmission disorders and long-term memory damage [8]. This suggests that this stage may be an important period for the accelerated deterioration of Aβ pathology. However, existing studies of mLVs damage in AD models rely exclusively on exogenous intervention methods such as photodynamic ablation [9–11]. Although these studies have confirmed that mLVs ablation can aggravate Aβ deposition and induce oligodendrocyte abnormalities [9] and aggravate neurological damage after viral infection [10], it is difficult to truly reflect the progressive functional decline of mLVs in the natural course of AD in such artificial intervention models.

This study addressed a key scientific question: In the absence of exogenous intervention, do AD mice models show spontaneous mLV drainage dysfunction as Aβ pathology progresses? To answer this question, we systematically compared the dynamic changes in mLV function between 3-, 6-, and 9-month-old APP/PS1 mice and wild-type (WT) controls. We focused specifically on the pathological turning point at 6 months of age to examine the age characteristics of brain lymphatic system dysfunction in the natural course of AD and its potential causal relationship with Aβ deposition.

## 2. Materials and methods

### 2.1. Animals

Male C57 BL/6 and APP/PS1 mice at 3, 6, and 9 months of age (n = 4 per group) were obtained from Changzhou Cavens Biotechnology Company Ltd. (Changzhou, China) (animal production license: SCXK (Su) 2020-0009, animal use license: SYXK (Su) 2023-0077). All mice weighed 25–32 g at the start of the experiments.

Animals were housed under specific pathogen-free conditions in the Animal Center of Nanjing University of Chinese Medicine, where they were maintained at 22–26 °C with 40–60% humidity and a 12/12 h light/dark cycle. Mice had ad libitum access to food and water, with regular bedding changes. Following 7 days of acclimatization, all experimental procedures were performed.

### 2.2. Behavioral experiments

The Morris water maze (MWM) uses a circular pool (120 cm in diameter and 60 cm in height) filled with milk powder water (22 ± 1 °C) to assess spatial learning and memory in rodents. During 5 days of acquisition training (4 times per day), mice used distal cues to locate a submerged platform (10 cm in diameter), and the trial was terminated after landing on the platform or after 60 s. Guidance was provided if necessary. A 60-s probe trial was performed 24 h after training, and memory retention was quantified by comparing the time to reach the platform. Data were collected using TopView behavior analysis tracking software, including escape latency and path length.

### 2.3. Intracisterna magna injection

After the mice were anesthetized with 1% sodium pentobarbital (50 mg/kg, intraperitoneal injection), they were depilated and fixed on a stereotaxic instrument; the muscle layer was peeled off to expose the cerebellomedullary cistern. The fluorescent tracer (Texas Red dextran 3, TR-d3, D3328, Thermo Fisher Scientific, Waltham, MA, USA) was dissolved in phosphate-buffered saline (PBS) at a concentration of 0.5%. Using a microinjection needle equipped with a 30 GA needle, 10 μL of the tracer solution was injected into the cerebellar cistern at a rate of 1 μL/min through a syringe pump. The injection needle was left in place for 5 min to prevent backflow; the mice were allowed to recover on a heating pad for 30 min.

### 2.4. Sample collection

The heart was perfused 30 min after intracisterna magna (ICM) injection. The mouse chest cavity was opened to fully expose the heart. The perfusion needle was inserted from the apex of the left ventricle, and PBS was perfused slowly and evenly until the blood color of the lungs and limbs faded and turned white. After the perfusion was completed, the neck hair was shaved, and a midline incision of about 3 cm was made on the neck about 5 mm above the clavicle. The superficial fat and fascia were bluntly separated, and the left sternocleidomastoid muscle was pulled to expose the left deep cervical lymph nodes (dCLNs). The lymph nodes were removed under a stereomicroscope. The right dCLNs were removed using the same method and frozen at −80 °C. Secondly, the skull was removed by decapitation, and scissors were used to cut off one circle along the lower edge of the parietal bone from the foramen magnum. The skull and brain tissue were carefully separated to obtain a complete skull top. The removed skull top was fixed in 12% paraformaldehyde for 24 h. Finally, the brain tissue was separated and fixed in 4% paraformaldehyde fixative at 4 °C for 24 h, embedded in paraffin, and sliced.

### 2.5. Immunohistochemistry

Tissue slides were baked on a slide oven at 60 °C for 1 h, dewaxed with xylene, rehydrated, and washed 3 times with PBS. The sections were immersed in antigen retrieval solution, microwave heated until boiling, and maintained for 10 min. The sections were cooled naturally to room temperature, then incubated with goat normal serum (Solarbio, Beijing, China, catalog number: SL038) at 37 °C for 20 min to block nonspecific binding, and then incubated with APP/beta amyloid polyclonal antibody (1:200, Proteintech, Chicago, IL, USA, catalog number: 25524-1-AP) at 4 °C overnight. The samples were then incubated with HRP-labeled goat antirabbit IgG at 37 °C for 20 min. Staining was performed using 3,3-diaminobenzidine. Finally, the sections were mounted with neutral gum to avoid bubbles prior to observation under a microscope and image collection.

### 2.6. Immunofluorescence

dCLNs were cut into coronal frozen sections, unfolded on slides, fixed, and blocked. Samples were incubated with the primary antibodies: anti-LYVE-1 antibody (1:200, Proteintech, catalog number: 51011-1-AP) and APP/Beta amyloid polyclonal antibody (1:200, Proteintech, catalog number: 25524-1-AP). The next day, the sections were labeled with the corresponding fluorescent antibodies. After incubation with DAPI, the sections were mounted. The dura mater was separated from the skull under a stereomicroscope, and the separated meninges were rinsed in PBS and incubated in PBS containing 2% bovine serum albumin and 0.3% Triton-X-100. Subsequently, the meninges were incubated with anti-LYVE-1 antibodies overnight and stained with fluorescent secondary antibodies the next day. The meninges were then carefully spread on slides and mounted with antifluorescence attenuation mounting medium.

### 2.7. Brain tracers

The hippocampus and cortical brain slices were sealed with a sealing solution containing DAPI, then scanned and photographed under a fluorescence microscope.

It should be noted that tracer penetration in brain parenchyma provides an indirect assessment of cerebrospinal fluid–interstitial fluid (CSF–ISF) exchange efficiency and does not constitute direct measurement of glymphatic function.

### 2.8. Ethical approval

This study was approved by the Experimental Animal Ethics Committee of Nanjing University of Chinese Medicine (approval number: 202404A052). All procedures were conducted in accordance with the relevant guidelines and regulations.

### 2.9. Statistical analysis

The imaging results were analyzed using ImageJ software for average fluorescence intensity and area. SPSS software version 27 was used for statistical analysis. First, the homogeneity of variance was checked and if applicable, analysis of variance was used to analyze the data. To compare the differences between groups, Tukey’s posthoc test was used. A p-value less than 0.05 was considered statistically significant.

## 3. Results

### 3.1. Behavioral results

**Figure 1:**
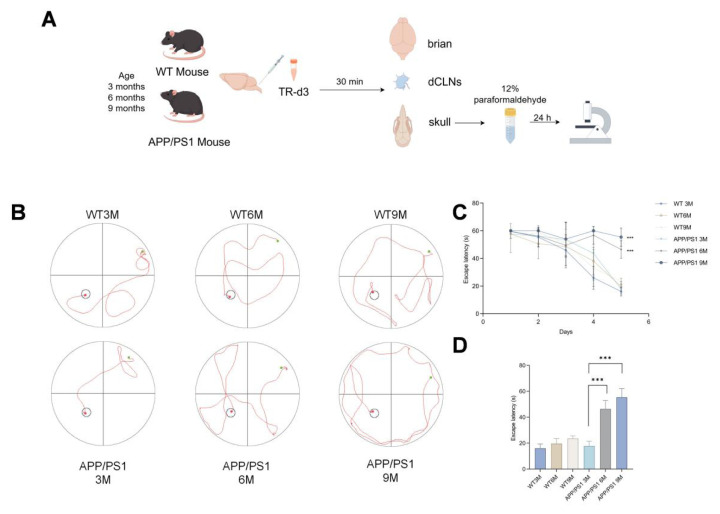
A) Schematic diagram of the experimental procedure, B) trajectory diagram of mice in each group in the MWM platform test, C) line graph of water maze escape latency, and D) escape latency on the fifth day (^***^ p < 0.001).

To systematically evaluate the spatial learning and memory capabilities of APP/PS1 mice at different ages, an experimental procedure was established as shown in Figure 1A, including the ages of the mice, ICM injection, and tissue collection time points. Behavioral results showed that the latency to reach the platform in the MWM significantly increased in 6-month-old and 9-month-old APP/PS1 mice (Figure 1B). As the number of training days increased, the escape latency of WT mice and 3-month-old APP/PS1 mice in each group gradually decreased (Figure 1C). After 5 days of learning and training, there was a significant difference in the escape latency between 6-month-old APP/PS1 mice and 9-month-old APP/PS1 mice compared to 3-month-old APP/PS1 mice (p < 0.001) (Figure 1D).

### 3.2. Age-dependent Aβ pathology emerged in APP/PS1 mice

**Figure 2:**
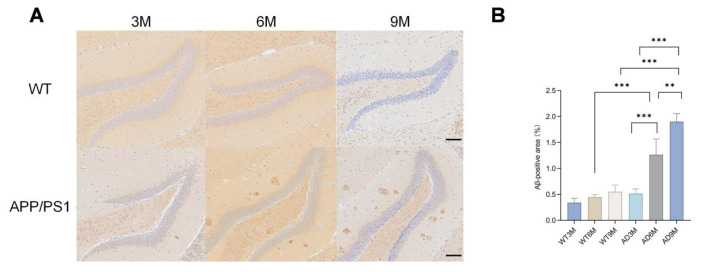
A) Immunohistochemistry of hippocampal sections. B) Aβ plaque area in different groups (^**^p < 0.01, ^***^p < 0.001).

No significant Aβ positive staining signals were detected in 3-, 6-, or 9-month-old WT mice and the 3-month-old AD model group (APP/PS1); however, a large amount of Aβ pathological deposition was found in the brain parenchyma of 6- and 9-month-old AD model mice (Figure 2B).

### 3.3. Progressive structural and functional decline of dCLNs in 6-month-old APP/PS1 mice

**Figure 3:**
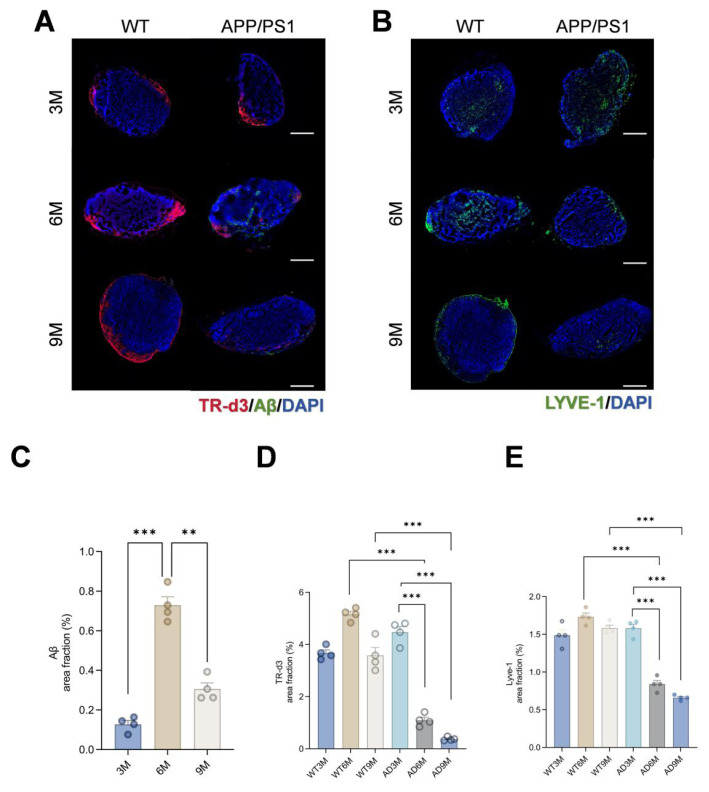
A) Representative images of TR-d3 tracer drainage and Aβ deposition in dCLNs, B) representative images of immunofluorescence of LYVE-1 in dCLNs, C) Aβ immunofluorescence area percentage in AD model mice, D) TR-d3 area percentage, and E) LYVE-1 immunofluorescence staining area percentage (^**^p < 0.01, ^***^p < 0.001).

Dynamic analysis of tracer transport in dCLNs showed a significant age-dependent reduction in the tracer-positive area in APP/PS1 mice (Figure 3A). Aβ immunofluorescence staining detected Aβ deposition in the dCLNs of 6-month-old APP/PS1 mice for the first time, with a notable decrease in deposition area in the 9-month-old group compared to the 6-month-old group (Figure 3A). LYVE-1 immunofluorescence staining showed progressive structural impairment of lymphatic vessels in the dCLNs of APP/PS1 mice with aging (Figure 3B).

Quantitative analyses further validated these observations. The area percentage of Aβ deposition in dCLNs initially increased in 6-month-old APP/PS1 mice but decreased in the 9-month-old group (Figure 3C), a pattern that may be associated with compensatory limitations in Aβ efflux resulting from lymphatic vessel structural damage. In contrast, the TR-d3 area percentage was significantly lower in both 6- and 9-month-old APP/PS1 mice compared to age-matched WT controls (Figure 3D). Furthermore, the LYVE-1-positive area percentage significantly decreased with age in APP/PS1 mice (Figure 3E) (p < 0.001). These findings indicate that APP/PS1 mice had progressive structural damage in dCLNs, which correlates with reduced Aβ clearance efficiency. Dysfunction of dCLNs may play an important role in the pathological process of AD.

### 3.4. Meningeal lymphatic dysfunction correlates with impaired tracer clearance in APP/PS1 mice at 6 months

**Figure 4:**
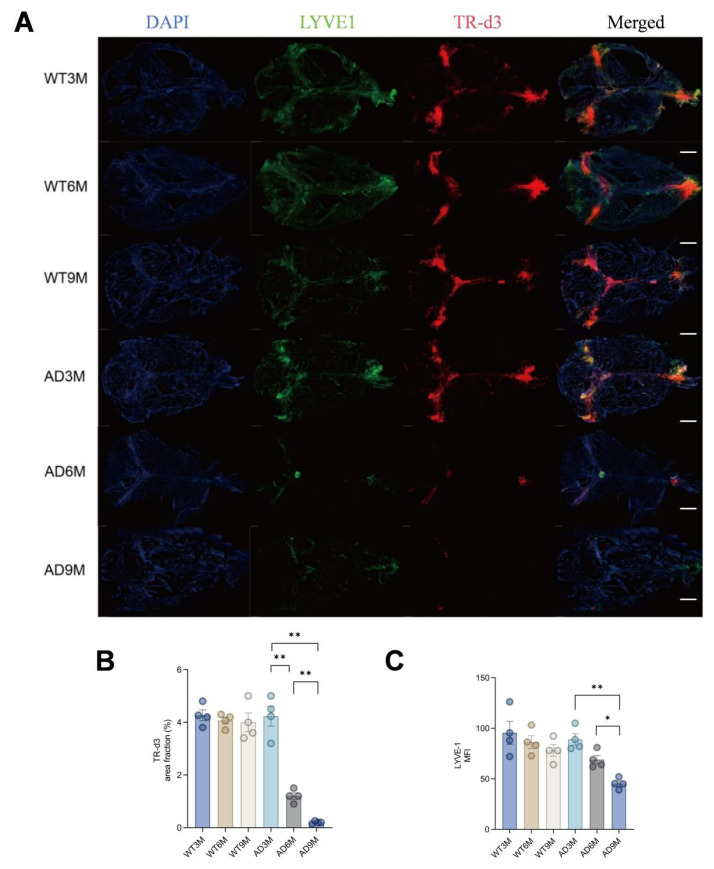
A) Representative images of mLVs, B) TR-d3 area percentage, C) LYVE-1 immunofluorescence mean intensity (^*^p < 0.05, ^**^p < 0.01, ^***^p < 0.001).

Dynamic evaluation of mLV drainage function in WT and APP/PS1 transgenic AD model mice was assessed by tracer injection (Figure 4A). Compared with the WT control group of the same age, the tracer fluorescence area in the mLVs of AD model mice showed an age-dependent decrease, indicating that the mLV-mediated metabolite drainage function was progressively impaired. Specifically, there was no significant difference in the tracer level between the 3-month-old AD group and the WT group of the same age, while the tracer in the mLVs of the 6-month-old and 9-month-old AD groups was significantly lower than that of the corresponding WT groups (p < 0.001), indicating that mLV drainage dysfunction first appeared at 6 months of age and further worsened at 9 months of age in the AD model mice (Figure 4B). The mLV tracer area remained stable across ages in WT groups, and no age-related functional decline was observed, suggesting that AD pathology has a specific effect on lymphatic drainage function. The mean fluorescence intensity (MFI) of LYVE-1 (a lymphatic endothelial marker) in mLVs decreased with age in AD model mice (Figure 4C), suggesting the structural integrity of mLVs may have been directly related to functional defects. The above evidence suggests that the progressive structural damage of mLVs in APP/PS1 mice (manifested by reduced LYVE-1 expression) may have been the pathological basis for the progressive decline in their drainage function, and that meningeal lymphatic dysfunction may have aggravated AD-related pathological processes by hindering the clearance of metabolic waste products (such as Aβ) in the brain.

### 3.5. Compromised CSF–ISF exchange efficiency in the hippocampus and cortex of APP/PS1 mice

**Figure 5:**
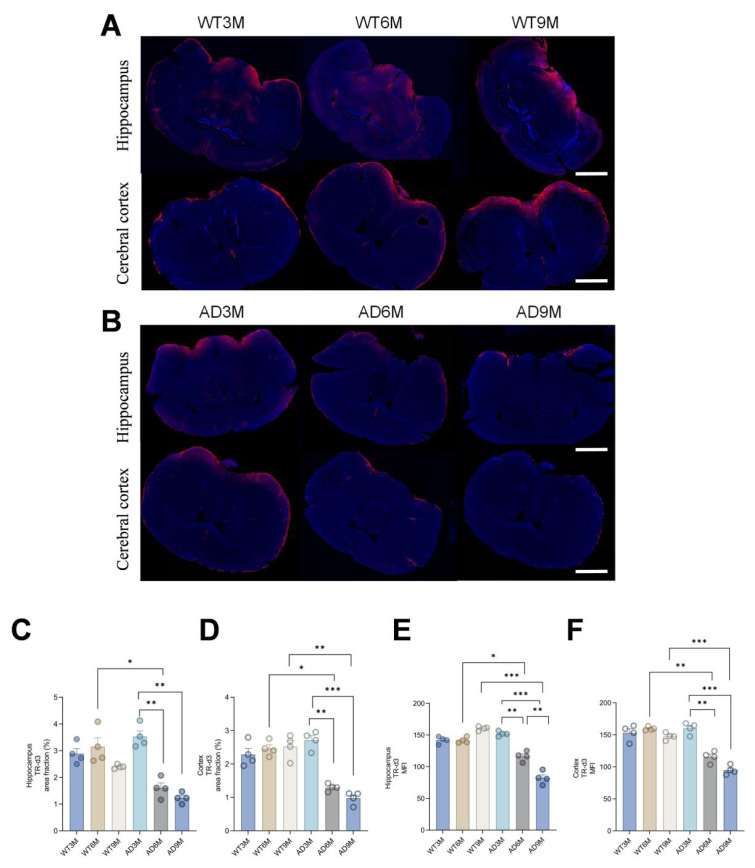
A) Distribution of TR-d3 in cortical and hippocampal slices of WT mice, B) distribution of TR-d3 in cortical and hippocampal slices of APP/PS1 mice, C) percentage of tracer area in hippocampal slices, D) percent of tracer area in cortical sections, E) MFI of tracers in hippocampal slices, F) MFI of tracers in cortical sections (^*^p < 0.05, ^**^p < 0.01, ^***^p < 0.001).

By injecting fluorescent tracers into the cisterna magna of WT and APP/PS1 transgenic AD model mice, brain tissue penetration efficiency was quantitatively analyzed (Figures 5A and 5B). The results showed that there was no significant difference in the tracer penetration rate in the hippocampus and cortex of mice in the WT group and the 3-month-old AD group; however, compared with the WT group of the same age, the tracer penetration rate in the hippocampus and cortex brain slices of the 6-month-old and 9-month-old AD group mice was significantly reduced (p < 0.01) (Figures 5C and 5D). The MFI of this area also synchronously decreased (Figures 5E and 5F), indicating that the CSF–ISF exchange function of AD model mice was progressively impaired from 6 months of age.

## 4. Discussion

This study systematically showed for the first time the spontaneous functional decline of the meningeal lymphatic system and dCLNs with age in APP/PS1 transgenic AD model mice without exogenous intervention, and its temporal correlation with the progression of Aβ pathology. The main findings can be summarized as follows: 1) the lymphatic drainage function of mLVs and dCLNs in 6-month-old AD mice significantly declined, evidenced by reduced tracer transport efficiency and a decrease in the LYVE-1 positive area; 2) structural disruption of mLVs (reduced LYVE-1 expression) occurs synchronously with dysfunction of CSF–ISF exchange, suggesting a potential functional link; and 3) the functional decline of the brain lymphatic system occurred simultaneously with the significant deposition of Aβ plaques (6 months of age) and was temporally correlated with the subsequent pathological deterioration (9 months of age), suggesting a potential role in AD progression associated with impaired Aβ clearance.

Through dynamic analysis at multiple time points (3, 6, and 9 months of age), this study found that the dysfunction of the lymphatic system in AD mice was dependent on age: normal function at 3 months of age, first significant damage at 6 months of age, and further deterioration at 9 months of age. This is highly consistent with the typical pathological explosive growth of Aβ plaques in APP/PS1 mice at 6 months of age [7]. The lymphatic function of WT mice remained stable at different ages, further indicating that AD pathology has a specific damaging effect on the lymphatic system. This finding extends the brain lymphatic system theory proposed by Louveau et al. [1], and offers new insights into impaired toxic protein clearance in AD. Studies have shown that the decrease in LYVE-1 expression (structural damage) and the decrease in tracer drainage efficiency (functional defect) in AD mice occur synchronously at 6 months of age, and Aβ deposition increases significantly thereafter.

This correlation suggests that the lymphatic system may be involved in the pathological process of AD, potentially through a proposed 3-level cascade hypothesis of structural damage leading to functional compensation failure and accumulation of toxic products. Recent studies have shown that borneol can improve Aβ clearance by enhancing mLV valve plasticity [12], indirectly verifying the importance of structural intervention for functional recovery. In addition, progressive damage to dCLNs may further weaken peripheral clearance capacity, potentially forming a vicious cycle of central–peripheral double defect. This is highly consistent with the lymphatic system network coordinated clearance model proposed by Da Mesquita et al. [13], but this study provides the first evidence of the temporal dynamic characteristics of this process in a natural course model.

Furthermore, while our data show a synchronous onset of significant lymphatic decline and Aβ plaque deposition at 6 months, the precise temporal sequence warrants consideration. It is plausible that the initial impairment of meningeal lymphatic function begins slightly prior to the detectable accumulation of Aβ, potentially even contributing to the tipping point for its aggregation. This interpretation is consistent with the intact lymphatic system and absence of Aβ pathology we observed at the 3-month presymptomatic stage. Should lymphatic decline indeed precede overt Aβ pathology, it would further substantiate the critical importance of the 6-month window for preemptive therapeutic intervention aimed at preserving lymphatic clearance capacity.

This study found that 6 months of age is the initial period of the decline in the lymphatic system function in AD mice, which provides a key window for early intervention. For example, targeted mLV-promoting lymphocyte-stimulating therapy such as VEGF-C administration [14] may block the pathological cascade reaction at this stage. In addition, the APP/PS1 model reproduces the progressive lymphatic damage characteristics of human AD in the natural course of the disease. Compared with traditional ablation models, it can more realistically simulate the development of the disease and provide an ideal platform for mechanistic research and drug screening.

The interpretation of our findings should consider several limitations inherent in our study design. Importantly, these limitations help to define clear and critical avenues for future research. It is important to note that the observational nature of our study, while capturing the spontaneous progression of the disease, means that the findings are correlational and do not prove causation. In addition, the sample size (n = 4 per group), though consistent with similar neurobiological studies, may limit the generalizability of findings. The exclusive use of male mice warrants consideration of potential sex-specific effects. While we used well-established immunohistochemical methods for Aβ detection, complementary biochemical analyses (e.g., ELISA) could further strengthen the robustness of Aβ quantification. Finally, our assessment of CSF–ISF exchange through tracer penetration provides an indirect measure of fluid dynamics and does not constitute a direct evaluation of glymphatic function, which should be addressed in future studies using specific methods.

Building upon these findings and addressing the current limitations, we propose the following directions for further investigation: 1) intervention studies (e.g., using VEGF-C to enhance mLV function) to establish a causal link between lymphatic repair and Aβ clearance, 2) including a wider age gradient (such as 12 months) to determine whether the functional decline persists, and 3) elucidating the role of basal mLVs to complement the dorsal mLVs[15] in Aβ clearance.

## 5. Conclusion

This study showed that APP/PS1 transgenic AD mice showed progressive structural and functional damage to the mLVs and dCLNs from 6 months of age without exogenous intervention, manifested by decreased LYVE-1 expression and reduced tracer drainage efficiency. There was also impaired CSF–ISF exchange. This phenomenon was significantly temporally correlated with the progression of Aβ pathology (explosive plaque deposition at 6 months of age). This study provides the first evidence linking spontaneous brain lymphatic decline to early Aβ deposition in AD. The 6-month age represents a potential early intervention window, coinciding with initial functional decline. The unique advantages of the APP/PS1 model in simulating the defects in AD lymphatic clearance function further highlight its value in mechanistic research and therapeutic strategy formulation.
